# An Innovative Day Hospital Dedicated to Nursing Home Resident: A Descriptive Study of 1306 Residents Referred by their Physicians

**DOI:** 10.1007/s12603-018-1106-5

**Published:** 2018-09-19

**Authors:** Clarisse Laffon de MaziÃ¨res, M. Romain, S. HermabessiÃ¨re, G. Abellan, S. Gerard, A. Castex, T. Krams, B. Vellas, Y. Rolland

**Affiliations:** 1 Department of Geriatric Medicine, GÃ©rontopÃ´le, Toulouse University Hospital (CHU de Toulouse), CitÃ© de la santÃ© - 20, rue du Pont Saint-Pierre - TSA 60033, 31059, Toulouse cedex 9, France; 2 Inserm UMR 1027, 37 AllÃ©es Jules Guesde, Toulouse, France

**Keywords:** Elderly, nursing home, emergency transfers, avoidable hospitalizations

## Abstract

**Background:**

The transfer rate of residents from nursing homes (NH) to emergency rooms is high. These transfers are often inappropriate but also potentially avoidable. Recent studies have shown that in terms of methods for training NH teams, proposals for improvement of the healthcare sector must be organized. Given this observation, GÃ©rontopÃ´le de Toulouse (France) opened in October 2015, a responsive day hospital dedicated to NH residents (DH NH). This day hospital is characterized by its vocation, exclusively dedicated to NH residents and its ability to provide patient care within a short period of time.

**Objectives:**

The purpose of this day hospital is twofold: (1) decrease the rate of inappropriate transfers for NH residents by offering general practitioners and NH teams quick access to expert advice, blood tests and radiological examinations during hospitalizations and care adapted to the characteristics of NH residents; (2) potentially reduce avoidable transfers to emergency rooms and hospitalizations by taking action to prevent acute decompensation in residents, but also for the education and training of NH healthcare teams. This manuscript aims to describe the arrangements put in place and the characteristics of the residents collected after two years of activity.

**Design:**

Retrospective descriptive study.

**Setting:**

GÃ©rontopÃ´le of Toulouse, France.

**Participants:**

1306 residents have been consulted at the DH NH.

**Measurements:**

Referring physicians (treating physicians, coordinating physician or emergency room physicians) send a standardized hospitalization request form to the day hospital by fax or email indicating the reason for the request, specialist opinion(s) desired and additional required examination(s). A gerontological assessment was conducted and anamnesis data was collected for each resident, on the very day of their coming to the DH NH.

**Results:**

In 2 years, 1306 residents from 120 NHs were sent to the DH NH. The mean age was 86.23 Â± 7.05 years and the majority of patients were women (n=941, 72.22%), dependent (median ADL at 2.75, [1.25-4.5]) and malnourished (821, 63.25%). In the 3 months prior to their visit to the day hospital, 668 (57.14%) residents had been hospitalized, and one-quarter (n=336, 25.72%) had been transferred to emergency rooms. The main reasons for hospitalization included assessment of cognitive disorders (n=336, 17.52%), assistance in managing behavioral disorders (n=297, 15.48%) and bedsores and slow wound healing (n=223, 11.63%).

**Conclusion:**

Our experience over a 2-year period suggests that the DH NH could be a practical response to the problem of inappropriate and avoidable transfers of NH residents to emergency rooms. This innovation could easily be utilized in other hospitals.

## Introduction

The transfer rate of residents from nursing homes (NH) to emergency rooms is high. In the United States, 25% of residents are transferred to emergency rooms at least once per year and 10% of residents 2 or more times (1). In France, approximately 50% of residents are hospitalized each year and more than half of the hospitalizations take place after a transfer to emergency rooms (2). In the United States, each resident is hospitalized nearly 2 times per year (3). These data reflect the great vulnerability of NH residents, but they also generate concerns because the transfer of NH residents to emergency rooms exposes them to multiple risks, such as confusion, falls, bedsores, functional decline, and mortality (4, 5, 6). In addition, these transfers cause organizational dysfunction within emergency units, which are often overcrowded and ill-equipped to care for elderly, dependent and often mentally ill subjects. Trips to the emergency room are often longer for NH residents than those of young subjects (7, 8, 9, 10).

Limiting the use of emergency rooms to patients who need it would be an unacceptable missed opportunity. However, many studies show that in 20 to 67% of cases the transfer of residents are potentially avoidable (11, 12, 13, 14, 15). Some data reports that more than two-thirds of residents have no severe clinical sign upon arrival to emergency rooms resulting in more than one-half of transfers to emergency rooms not requiring subsequent hospitalization (3). In addition, nearly 20% of residents leave without any diagnosis. These data suggest that alternatives must be organized to limit the number of potentially avoidable transfers to emergency rooms.

INTERACT (Interventions to Reduce Acute Care Transfers), a program for the improvement of clinical practices, was arranged in the United States in order to limit the transfer of NH residents to hospitals. This program, which is based on improving the identification, assessment and management of acute medical situations by the NH healthcare team, has not yet demonstrated its efficacy on decreasing hospitalizations (16). The results of INTERACT suggest that in terms of training NH teams, proposals for improving the healthcare sector must be organized for residents, because despite the actions carried out within the NH, the healthcare teams often remain confronted with acute medical situations requiring additional tests and expert advice. The risks associated with transfers to emergency rooms are well known to treating physicians and NH healthcare teams, but the lack of quick response from traditional hospital services or other alternatives often results in an inappropriate use of emergency rooms (11, 17). To date, no other sector offers the simplicity and rapid access to technical platforms, expert advice and monitoring of emergency services. However, in the IQUARE study, we have shown that collaboration between the NH healthcare team and geriatricians decreases the number of emergency room transfers for residents (18). Other studies also show that apart from the characteristics of residents (multiple comorbidities (19), degree of dependence), the organization within the NH (care protocol, partnership with the hospital) determines the transfer rate of residents to emergency rooms (17, 20).

Given this observation, GÃ©rontopÃ´le de Toulouse (France) opened in October 2015, a responsive day hospital dedicated to NH residents (DH NH). This day hospital is characterized by its vocation, exclusively dedicated to NH residents and its ability to provide patient care within a short period of time. The purpose of this day hospital is twofold: (1) decrease inappropriate transfers to emergency rooms by offering general practitioners and NH teams quick access to expert advice, blood tests and radiological examinations during hospitalizations and care adapted to the characteristics of NH residents; (2) reduce potentially avoidable transfers to emergency rooms and hospitalizations by taking action to prevent acute decompensation in residents, but also for the education and training of NH healthcare teams.

In this article, we present the organization of the responsive day hospital dedicated to NH residents and the main characteristics and reasons for sending residents collected since its opening.

## Method

The innovative day hospital dedicated to NH residents occupies a central position within the care units of the Geriatrics Department of Toulouse, located in the Centre Hospitalier Universitaire (CHU) de Toulouse (France). The admission capacity is approximately 4 residents per day. The capacity of the home has increased in one year to 6 residents per day, 5 days per week. A full-time geriatrician is on staff at the day hospital as well as other medical specialists, who are on-call and respond to requests as needed. Nurses work at a rate of 1.8 full-time equivalents (FTE) (i.e. 35 hours/week) and caregivers at 1 FTE. Nurses provide preventive, curative or palliative care to residents and are also involved in scheduling examinations, which will benefit the residents during their hospitalization as well as the convening of residents. The premises consist of one room per patient (bed and bathroom), a treatment room, scheduling office and medical office.

The methods of sending to the day hospital are according to protocol. Residents can be directed to the DH, either by the physician (treating physician or coordinating physician), or by emergency room physicians after transferring to the emergency room (post-emergency room care).

In practice, the requesters (treating physicians, coordinating physician or emergency room physicians) send a standardized hospitalization request form to the day hospital by fax or email indicating the reason for the request, specialist opinion(s) desired and additional required examination(s) (biological and/or radiological). Mention of the urgent nature of care is explicitly requested on this form in order to prioritize the requests. Request forms may be accompanied by a telephone call to the day hospital physician, particularly for urgent requests (hospitalization request within 48â€“72 hours) in order to the fulfill the request and accelerate the treatment. The request form is reviewed by the geriatrician at the day hospital. After approval of the request by the DH physician and obtaining of any additional information, the nurse organizes the resident's hospital day by contacting the various specialists required and making appointments for additional examinations. Finally, the nurse contacts the NH to communicate the date for the resident to come to the day hospital.

This procedure allows emergency room physicians to see NH residents who do not require traditional hospitalization after having been examined in the emergency room, but who could benefit from a geriatric opinion and/or another specialist as well as any additional examinations. This procedure enables early reorientation of residents to their NH after a short stay in the emergency room without having to wait for additional examinations, which are not performed in the emergency room.

Various opinions of medical specialists may be given at the day hospital (neurologist, cardiologist, urologist, pulmonologist, psychiatrist, rheumatologist, physical therapist, botulinum toxin specialist, infectologist, geriatric oncologist, palliative care/ pain specialist, specialist in bedsores and slow wound healing, dental surgeon). This singular service can be carried out in the resident's room without having to be transferred. In other words, the specialist visits the resident's bedside in the DH (for example, the heart ultrasound is performed in the resident's room in the DH NH) through the assistance of other physicians in the Geriatrics Department, but also in partnership with the physicians of other hospital departments who are aware of the constraints caused by moving dependent and often mentally ill residents. Medical imaging (CT and other scans) is carried out in a nearby building. Preferred time slots for X-rays have been defined with the Radiology Department in order to limit appointment delays.

Moreover, residents can benefit from other hospital services like any other hospitalized patient.

An organization is in place to ensure that the expertise of paramedics is available. When residents come to the DH, they can benefit from the advice of a dietitian, an occupational therapist, a physiotherapist, and a speech therapist. Finally, the prescriptions of all residents are systematically reviewed by a pharmacist with an expertise in geriatrics. Proposals for prescription changes are systematically included in the discharge letter.

In order to modify the practices, an information campaign on this new activity was organized among the regional actors of geriatric care. Between March and October 2015, coordinating physician and/or coordinating nurse in NHs of the Toulouse healthcare field were notified by telephone of the opening of this day hospital and in traditional hospital discharge letters for residents when they returned to their NH or when they entered into a NH. Regionally, this new activity was the subject of an oral presentation at a regional congress (scientific meetings on aging with approximately 300 physicians and nurses). Nationally, we presented the 1-year assessment of this innovation at the CongrÃ¨s National des UnitÃ©s de Soins, d'Ã©valuation et de prise en charge des patients Alzheimer (USPALZ) and the 36th JournÃ©es Annuelles de la SociÃ©tÃ© FranÃ§aise de GÃ©riatrie et GÃ©rontologie.

The characteristics of all residents sent to the DH NH during the first two years of activity were systematically entered in a standardized and prospective manner: data was collected for each resident, on the very day of their coming to the DH NH. The data collected were: age, sex, Charlson comorbidity index (21), degree of dependency assessed by the activities of daily living (ADL) scale on the hospitalization day and 3 months before (a score of 6 indicating total autonomy and 0 total dependence) (22). With the collection of weight (kilogram), height (meter), and serum albumin levels (gram/ liter), the nutritional status (malnutrition, yes/no) of each resident was defined according to the criteria of the Haute AutoritÃ© de SantÃ© (23). The ability to walk was collected as a categorical variable (alone, with human assistance, with technical assistance, no ability to walk), as well as the presence of a sensory deficit (visual, auditory, auditory and visual, no deficit), falls during the month (yes/no), presence of pain (yes/ no) and bedsores (yes/no). The number of prescription drugs was analyzed as a continuous variable and the prescription of at least one neuroleptic drug (yes/no), benzodiazepine (yes/ no), or antidepressant (yes/no) was noted. The MMSE score was collected. The presence of behavioral disorders (yes/no) and, if applicable, the Neuropsychiatric Inventory (NPI) (24) score were collected. The Mini-Geriatric Depression Scale was analyzed as a continuous variable (a score of 0 indicates the high probability of no depression, and â‰¥ 1 indicates a high probability of depression). Finally, we note whether the resident was sent directly by his/her treating physician at the day hospital (yes/no).

### Statistical analyses

Data collection and statistical analyses were made by the referring physician of the DH. The continuous variables were expressed by the mean and standard deviation (SD) (mean Â± SD) when they had a Gaussian distribution, otherwise by the median and deviation type (median [25-75]). Categorical variables were expressed in number and percentage. The analyses were carried out using Stata v14.2 software (StataCorp, College Station, TX, USA).

## Results

### Characteristics of the residents

The main characteristics of the 1306 NH residents who were sent to the innovative day hospital for NH residents over a 2-year period are presented in Table 1. The mean age was 86.23 Â± 7.05 years and they were mainly women (n=941, 72.22%). The residents were very dependent with a median ADL at 2.75, [1.25-4.5], and 821 (63.25%) were malnourished. In the 3 months prior to their visit to the day hospital, 668 (57.14%) residents had been hospitalized, and one-quarter (n=336, 25.72%) had been transferred to emergency rooms. 814 residents (66.67%) were sent directly by the treating physician to the DH NH.

**Table 1:** Description of the characteristics of residents who have been consulted at the innovative day hospital dedicated to nursing home residents

Characteristics (n = 1306)	Mean Â± SD or median [25-75] or n (%)
Age (y), n=1301	86.23 Â± 7.05
Gender (female), n=1303	941 (72.22)
ADL, n=1241	2.75 [1.25-4.5]
Loss of 1 or more ADL points in the last 3 months, n=429	86 (20.05)
Charlson comorbidity index, n=1086	3 [2-4]
Ability to walk, n=1202	
Yes, alone	343 (28.54)
Yes, with human assistance	162 (13.48)
Yes, with technical assistance	315 (26.21)
No	382 (31.78)
Fall during the month, n=1183	228 (19.27)
Weight, n=1282	61.87 Â± 14.41
BMI, n=1219	24.18 Â± 5.16
Albumin (g/l), n=1203	34.02 Â± 4.66
Malnutrition, n=1298	821 (63.25)
Sensory deficit, n=933	
xx	xx
Yes, auditory	183 (19.61)
Yes, visual	183 (19.61)
Yes, auditory and visual	150 (16.08)
No	417 (44.69)
Number of drugs, n=1295	8.04 Â± 3.24
At least one prescribing of :	
- Neuroleptic, n=1267	291 (22.95)
- Benzodiazepine, n=1282	745 (57.07)
Antidepressant, n=1274	665 (52.12)
MMSE score, n=640	15 [9-19]
BPSD, n=1201	513 (42.71)
NPI score, n=318	34 [20-51]
Hospitalization within 3 months, n=1169	668 (57.14)
Scheduled hospitalization	375 (28.71)
Hospitalization in emergency room	336 (25.72)
Presence of bedsores, n=1199	211 (17.60)
Pain, n=1217	159 (13.06)
Short-GDS, n=363	0 [0-2]
Addressed directly by treating physician, n=1221	814 (66.67)


ADL, Activities of Daily Living [0 = Low (patient very dependent), 6 = High (patient independent)]; BMI, Body Mass Index; GDS, Geriatric Depression Scale; g/l, gram per liter; MMSE, Mini Mental State; BPSD, Behavioural and Pyschological Symptoms of Dementia; NPI, Neuropsychiatric Inventory;

### Reasons for hospitalization

Table 2 presents the reasons for hospitalization. Thus, 1918 different reasons for hospitalization were collected or 1.5 reasons per resident. The main reasons for hospitalization included assessment of cognitive disorders (n=336, 17.52%), assistance in managing behavioral disorders (n=297, 15.48%) and bedsores and slow wound healing (n=223, 11.63%). Nutritional (n=197, 10.27%), neurological (n=193, 10.06%) and cardiac (152, 7.92%) reasons represented nearly one-third of the requests.

**Table 2:** Reasons for hospitalization of residents hospitalized in the innovative day hospital dedicated to nursing home residents

Reasons for hospitalization (n=1918)	n	%
Cognitive disorders	336	17.52
Behavioral disorders	297	15.48
Bedsores and slow wound healing	223	11.63
Nutritional status	197	10.27
Neurology	193	10.06
Cardiology	152	7.92
Psychiatry	71	3.70
Assessment of fall	65	3.39
Internal medicine	57	2.97
Transfusion	49	2.55
Urology	44	2.29
Pain and palliative care	33	1.72
Botulinum toxin injection and monitoring	25	1.30
Hematology	22	1.15
Occupational therapy	19	0.99
Physical therapy	15	0.78
Pneumology	14	0.73
Reassessment after acute episode	12	0.63
Rheumatology	12	0.63
Dentist	12	0.63
Speech therapist	10	0.52
Endocrinology	9	0.47
Geriatric oncology	7	0.36
Gastroenterology	7	0.36
Other reason	37	1.93

### Reactivity

The time period of care was not collected for all 1306 residents. However, for 20 residents, chosen at random, for whom the treating physicians had mentioned the urgent nature of the request, the time period of care was 2.7 days.

The occupancy rate increased 77% in the opening year to 92.3% in 2017 and was 93.2% from January to March 2018. The number of NHs in the region that sent residents to the DH NH was 120 and among them, 80 sent residents to the DH NH several times.

## Discussion

Reducing the inappropriate use of the emergency room for NH residents while meeting the expectations of caregivers faced with caring for residents is an important mission that hospital geriatric teams must assume. To our knowledge, our proposal of the responsive day hospital specifically dedicated to NH residents is a new innovative alternative that offers a practical response to the problem of inappropriate and potentially avoidable hospitalizations for NH residents.

Without demonstrating the efficiency of our system, our experimentation over a 2-year period suggests that, in many clinical situations, residents can be cared for without going to the emergency room and without being exposed to hospital iatrogenics (25). The occupancy rate of this new structure and the high number of NHs that regularly send their residents to it also testifies to its usefulness for NH care teams. Without such a system, NHs struggle to care for a variety of clinical situations which result in transfers to emergency rooms, in the absence of an alternative (11). Decisions to transfer NH residents to emergency rooms most often involve a conscious medical choice. Previous works showed us that the decisions to transfer decisions are usually made by a doctor (72% of cases), and more often (65%) during the day (26). Care with a medical and paramedical team specializing in geriatrics, short care times, easy access to specialist advice and a technical platform, without multiplying the movements of residents, appear in our action as a realistic alternative to meet the expectations of actors in the field. Characteristics of residents sent to the DH NH (Table 1) correspond to those of dependent, polypathological, elderly patients, regularly and recently hospitalized and thus at high risk of new transfers to emergency rooms. The reasons for sending patients are very often associated with dementia complications (particularly psychobehavioral disorders) and are problems primarily faced by NHs (2). The reasons correspond well to factors for inappropriately sending residents to emergency rooms suggesting that they have somatic decompensation falling within the emergency category and have been successfully transferred to emergency rooms. In other words, we believe that our strategy of sending residents directly to this DH, which is the proper target of residents without causing missed opportunities. This is made possible by the organization of upstream care for emergency rooms, but also through a system allowing a response within short time periods.

The prevention of inappropriate hospitalizations for NH residents is a complex approach that can only be effective if it is based on multiple synchronized actions (25). The importance of training (27), motivation (28) of NH care teams, implementation of preventive measures, use of the assessment protocol before transferring to the emergency room (16), taking into account the opinion of the resident, drafting advance directives, and prior discussion with the families are all means which, in fine, help to curb the inappropriate use of emergency rooms. The INTERACT program developed in the United States focused on training the NH care team, but not involving all the levers of action, did not show its effectiveness by decreasing hospitalizations (16). Therefore, it seems important to associate these methods of training NH teams with an improvement of the healthcare sector for residents. A specificity of the DH NH is to provide care centered on the patient, adapted to the functional limitations of the resident, concerned about direct communication with the NH (all residents responded to the DH with a detailed letter by considering the quality of communication as an important factor in the flow of hospital-to-NH transitions) (29). This communication is also centered on preventing the most frequent causes of avoidable hospitalizations.

Thus, drug-induced iatrogenic disorder is a frequent cause of avoidable hospitalizations for NH residents (30). The study conducted by Lau et al. shows that residents who had at least one potentially inappropriate prescription had a significantly higher risk of hospitalization than residents who did not (OR, 1.27; p=0.002) (31). For all residents hospitalized in the DH NH, a hospital pharmacist made an analysis of their prescriptions. Proposals were given in the hospital discharge letter to improve the prescription in order to reduce druginduced iatrogenic disorder (32). We believe that this strategy has didactic virtues and therefore an impact that goes far beyond the single case of the assessed resident. The proposals aim to combat inappropriate prescriptions (overuse), allow for therapeutic adjustments (misuse), but are also the opportunity to give reminders of preventive measures (e.g. influenza and pneumococcal vaccine, vitamin D supplementation33) (underuse). The relevance of this prescription revision is optimized by direct collaboration between the geriatrician and the pharmacist present within the DH.

The DH NH is also a real asset within a partnership with emergency room physicians, who can then respond to the problem of early readmissions to emergency rooms by directing NH residents to a DH in the days that follow, when traditional hospitalization after the transfer to an emergency room is not required.

**Figure 1:**
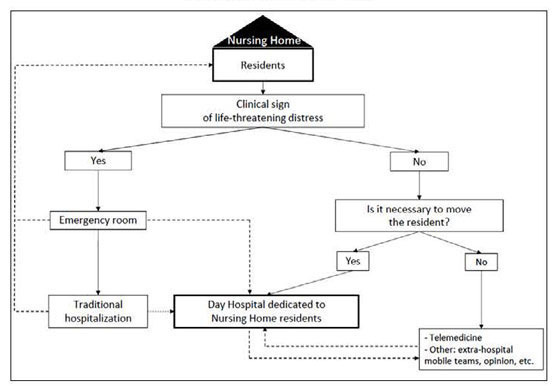
Day hospital dedicated to nursing home residents within geriatric care network

We can also only exclude a proportion of residents passing through the DH NH inappropriately. However, no resident was in a situation falling under the category of a vital emergency. Furthermore, we believe that an improper transfer to the DH NH would not have unfavorable consequences like an improper transfer to an emergency room. The DH NH must not be substituted for any other types of care, such as telemedicine especially when moving the resident is not justified (Figure 1). It would therefore be interesting, in a later study, to verify whether the hospitalizations of residents in the DH NH are appropriate.

In conclusion, our experience over a 2-year period suggests that the innovative day hospital for NH residents could be a practical response to the problem of inappropriate and avoidable transfers of NH residents to emergency rooms. Care centered on the resident and adapted to his/her characteristics, minimizes the iatrogenic events linked to traditional hospitalization and allows the quick redress to advice from specialists and a technical platform in a short period of time while not multiplying the movements of the resident. We believe that this innovation could easily be utilized in other hospitals.
